# Metabolic Reprogramming of Astrocytes in Pathological Conditions: Implications for Neurodegenerative Diseases

**DOI:** 10.3390/ijms25168922

**Published:** 2024-08-16

**Authors:** Corrado Calì, Iva Cantando, Maria Fernanda Veloz Castillo, Laurine Gonzalez, Paola Bezzi

**Affiliations:** 1 Department of Neuroscience “Rita Levi Montalcini”, University of Turin, 10124 Turin, Italy; maria.velozcastillo@kaust.edu.sa; 2 Neuroscience Institute Cavalieri Ottolenghi, 10143 Orbassano, Italy; 3 Department of Fundamental Neurosciences (DNF), University of Lausanne (UNIL), 1005 Lausanne, Switzerland; iva.cantando@unil.ch (I.C.); laurine.gonzalez@unil.ch (L.G.); 4 Biological and Environmental Science and Engineering Division, King Abdullah University of Science and Technology, Thuwal 23955-6900, Saudi Arabia; 5 Department of Physiology and Pharmacology, University of Rome Sapienza, 00185 Rome, Italy

**Keywords:** astrocyte, reactive astrocytes, metabolism, mitochondria, L-lactate, ketone bodies, fatty acid oxidation, Alzheimer’s disease, Huntington’s disease

## Abstract

Astrocytes play a pivotal role in maintaining brain energy homeostasis, supporting neuronal function through glycolysis and lipid metabolism. This review explores the metabolic intricacies of astrocytes in both physiological and pathological conditions, highlighting their adaptive plasticity and diverse functions. Under normal conditions, astrocytes modulate synaptic activity, recycle neurotransmitters, and maintain the blood–brain barrier, ensuring a balanced energy supply and protection against oxidative stress. However, in response to central nervous system pathologies such as neurotrauma, stroke, infections, and neurodegenerative diseases like Alzheimer’s and Huntington’s disease, astrocytes undergo significant morphological, molecular, and metabolic changes. Reactive astrocytes upregulate glycolysis and fatty acid oxidation to meet increased energy demands, which can be protective in acute settings but may exacerbate chronic inflammation and disease progression. This review emphasizes the need for advanced molecular, genetic, and physiological tools to further understand astrocyte heterogeneity and their metabolic reprogramming in disease states.

## 1. Introduction

The brain is one of the most metabolically active organs in the body. Despite comprising only about 2% of the total body weight, the brain accounts for a significant 20% of the resting total body oxygen consumption [1,2,3,4,5,6,7,8,9,10,11]. In children, especially during the first decade of life, the developing brain can utilize as much as 50% of the body’s oxygen consumption [8,12,13,14,15,16,17]. Similarly, the brain’s glucose consumption is substantial. The global cerebral metabolic rate of glucose represents about 20% of the resting total body glucose consumption in adults [18]. This rate peaks between the ages of 5 and 9 years, accounting for over 50% of the body’s resting metabolic rate during this period [8,15,16,17,19,20,21].

Astrocytes play a pivotal role in maintaining the brain’s energy balance. As the predominant and highly branched cell type in the central nervous system, astrocytes perform diverse functions, including the formation of the blood–brain barrier (BBB) through interactions with cerebral vessels. Additionally, perisynaptic astrocytic processes (or PAPs) form the third element in the “tripartite synapse” [22,23,24], actively modulating synaptic activity [25,26,27,28,29,30,31,32,33] and providing crucial metabolic support to neurons [8]. These glial cells sequester excess glutamate and potassium ions (K^+^) and release essential chemical messengers such as gliotransmitters, growth factors, lactic acid, and glutamine, thereby protecting neighboring cells [26,27,34,35,36,37].

Astrocytes are strategically located between capillaries and synapses, where they actively regulate neurometabolic and neurovascular couplings, establishing a crucial link between neuronal activity and brain energy consumption [8,20,21]. Glucose is an essential fuel for numerous brain functions, including ATP production, oxidative stress management, and the synthesis of neurotransmitters, neuromodulators, and cellular structural components. Experimental ratios between glucose and oxygen consumption at rest suggest incomplete glucose oxidation, potentially leading to significant lipid or amino acid production from glucose or the excretion of unoxidized metabolites, particularly L-lactate [8,38]. The brain’s substantial glycolytic capacity is likely attributed to astrocytes, where glycolysis appears to have a more prominent enzymatic capacity than oxidative metabolism [37]. Astrocytic glycolysis is enhanced by neurotransmitters like glutamate and noradrenaline, and the astrocyte-neuron L-lactate shuttle (ANLS) model proposes neuronal ATP production using astrocyte-derived L-lactate, which is implicated in activity-dependent energy metabolism and cognitive function [8,20].

Astrocytes are also crucially involved in lipid metabolism, producing lipids which are supplied to neurons and oligodendrocytes for synaptic and myelin membrane components [39]. Astrocytes are the main sites for fatty acid oxidation in the brain [40]. While lipid oxidative metabolism is limited compared with glucose in the resting brain, it is regulated by interactions between astrocytes and neurons [41,42,43]. Ketone bodies (KBs), which are major energy substrates in neonatal and postnatal brains, are also produced during energy deficits, and fatty acid oxidation and ketone bodies are essential in the brain as an alternate source of energy for maintaining normal brain functions [39,44,45,46,47,48,49,50]. Astrocytes are exceptionally capable of storing and oxidizing fatty acids, and local KB production and delivery within neighboring cells have been demonstrated recently in *Drosophila melanogaster* [50].

In recent years, a multitude of studies have highlighted the pivotal role of mitochondria in both developing and mature astrocytes for maintaining homeostatic functions and supporting associated synapses [51,52,53]. Although astrocytes primarily rely on glycolysis for their energy production [54,55,56], they are nonetheless responsible for approximately 20% of the brain’s total oxygen consumption [57]. This significant oxygen usage is predominantly associated with oxidative phosphorylation occurring in astrocytic mitochondria, which is essential to produce adenosine triphosphate (ATP). Astrocytic mitochondria play a crucial role in various cellular processes, ensuring the proper function and survival of astrocytes as well as the neurons they support.

This review aims to explore recent findings which shed light on the metabolic intricacies of astrocytes, particularly in the context of pathological conditions, and their potential significance in influencing the initiation of neurodegenerative diseases. By synthesizing emerging evidence, this review investigates the multifaceted roles which astrocytes play in the metabolic landscape of the central nervous system (CNS) during disease states. Understanding how astrocytic metabolism changes under pathological conditions provides crucial insights into the complex interplay between metabolic dysregulation and the onset of neurodegenerative diseases. This exploration may pave the way for novel therapeutic strategies targeting astrocyte-specific metabolic pathways, offering new avenues for intervention and potential breakthroughs in the management of neurodegenerative disorders.

## 2. Metabolism of Astrocytes in Physiological Conditions

**Figure 1:**
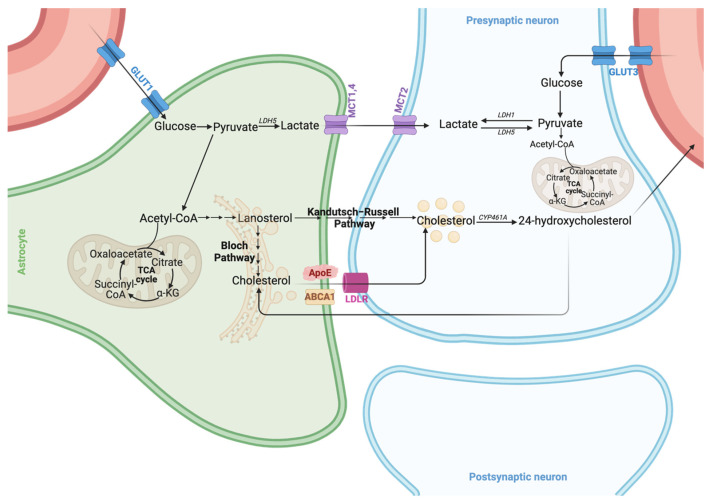
Glucose metabolism and cholesterol synthesis. Glucose enters the bloodstream and travels to the astrocytes and neurons via GLUT1 and GLUT3 transporters, respectively [64]. In astrocytes, lactate is produced as an end product of glycolysis and is shuttled via monocarboxylate transporters (MCTs) 1 and 4 and taken up by neurons via MCT2. Neurons possess the enzymatic machinery to convert lactate into pyruvate, which is then utilized to fuel the tricarboxylic acid (TCA) cycle. Cholesterol biosynthesis starts with the transport of Acetyl-CoA from the mitochondria to the cytosolic side of the endoplasmic reticulum, where lanosterol is produced. Then, cholesterol will be synthesized via the Bloch pathway, which occurs in the astrocytes, and via the Kandutsch–Russell pathway, which takes place in neurons. The cholesterol produced in the astrocytes is shuttled to neurons in ApoE-containing lipoproteins via ABCA1 transporters and taken up by receptor-mediated endocytosis via LDLR family receptors. Excess cholesterol is metabolized to 24-hydroxycholesterol, which crosses the BBB and passes into circulation to be eliminated through hepatic circulation. Much 24-hydroxycholesterol will be taken up by astrocytes to activate LXR and upregulate the expression levels of ApoE and ABCA1.

Astrocytes possess comprehensive metabolic capabilities, engaging in various aspects of glucose metabolism with a notable preference for glycolysis. Glucose uptake in astrocytes is facilitated by glucose transporters (GLUTs). Once inside the cell, glucose can either be stored as glycogen or enter various metabolic pathways. The primary pathway is glycolysis, which converts glucose to pyruvate and then to lactate via lactate dehydrogenase (LDH). This lactate is subsequently shuttled to neurons through monocarboxylate transporters (MCTs), where it serves as an energy source, supporting neuronal function and survival [38,58,59]. L-lactate can be alternatively synthesized via glycogenolysis, a pathway downstream of the activation of astrocytic β_2_-adrenergic receptors [60,61] which is necessary for learning and memory [62] as well as for structural synaptic stabilization [63]. Astrocytes’ substantial glycolytic capacity is mainly attributable to their unique gene expression profile, which favors converting pyruvate to lactate over utilizing it in the tricarboxylic acid (TCA) cycle (Figure 1).

This is facilitated by lower activity of the enzyme pyruvate dehydrogenase (PDH) in astrocytes due to high phosphorylation levels, promoting the aerobic glycolytic profile [65,66]. This preference for glycolysis, even under sufficient oxygen conditions, is known as aerobic glycolysis or the Warburg effect, which is crucial for neuroprotection and brain homeostasis. During neuronal excitation, astrocytic glycolysis is upregulated, leading to increased lactate production and release. This lactate functions as both an energy substrate and a signaling molecule, modulating neuronal excitability and promoting the expression of genes related to survival and plasticity [38,58,59,67]. Additionally, astrocytes channel glucose through the pentose phosphate pathway (PPP), generating NADPH and ribose-5-phosphate, which are essential for antioxidant defense and nucleotide synthesis. PPP activity in astrocytes is significantly higher than that in neurons, highlighting its role in protecting neurons against oxidative stress by providing reduced glutathione [58].

Astrocytes and neurons differ in the regulation of glycolytic enzymes [65] and in the organization of their mitochondrial respiratory chain [68]. Astrocytes rely on glycolysis for energy generation and, as a consequence, have a loosely assembled mitochondrial respiratory chain which is associated with greater generation of mitochondrial reactive oxygen species (ROS) [68]. While it is well established that mitochondria in astrocytes are a significant source of ROS, the specific physiological roles of these molecules have remained unclear. However, a recent study shed light on this issue, revealing that astrocytic mitochondrial ROS are crucial regulators of brain metabolism and neuronal function [69]. In this study, researchers generated mice which inducibly overexpressed mitochondria-targeted catalase in astrocytes, leading to decreased mitochondrial ROS production in these cells during adulthood. Comprehensive analyses, including transcriptomic, metabolomic, biochemical, immunohistochemical, and behavioral assessments, demonstrated that reducing mitochondrial ROS in astrocytes caused significant changes in the brain redox balance, as well as the carbohydrate, lipid, and amino acid metabolic pathways. These changes were associated with altered neuronal function and mouse behavior. Notably, the study found that astrocytic mitochondrial ROS play a critical role in regulating glucose utilization through the pentose-phosphate pathway and glutathione metabolism. This regulation in turn influences the redox status and potentially the survival of neurons, highlighting the essential role of mitochondrial ROS in maintaining neuronal health and function.

Astrocytes exhibit poor mitochondrial respiration, partly because of complex I uncoupling from supercomplexes, supporting the glycolytic pathway [46]. Consequently, mice with a loss of astrocyte mitochondrial respiration due to conditional Cox10 mutations were phenotypically normal, remaining without signs of neurodegeneration for over a year [47]. However, recent studies, which have involved sorting astrocytes from whole mouse brains, have begun to reveal that these cells not only exhibit high levels of glycolytic enzyme expression but are also enriched in various enzymes of the TCA cycle [70,71]. This finding supports the idea that astrocytes possess a significant oxidative capacity and highlights their ability to dynamically adjust their metabolic profile in response to substrate availability and energy demands [21,72]. Such metabolic flexibility underscores the unique capacity of astrocytes to adapt to extreme metabolic challenges, such as those encountered during ischemia, injury, and inflammation.

Recent advances have highlighted that mitochondria in astrocytes are not only critical for ROS production but also serve as key storage sites for Ca^2+^, playing a significant role in intracellular Ca^2+^ sequestration and signaling [73]. Elevations in cytosolic Ca^2+^ appear to promote the immobilization of mitochondria near highly active synapses [74] and fine processes where Ca^2+^ transients occur most frequently [75]. These mitochondria are strategically located to meet higher local energy demands [76], shape Ca^2+^ fluctuations [77,78], and modulate Ca^2+^-dependent processes such as gliotransmission [74,79,80].

Astrocytes also play a crucial role in lipid metabolism. They are responsible for the uptake and storage of various lipids, including fatty acids and cholesterol, which are vital for maintaining neuronal function and membrane integrity [81,82]. Astrocytes synthesize and secrete cholesterol, which is essential for forming and maintaining myelin sheaths around axons [83]. Cholesterol synthesis starts with the transport of acetyl-coenzyme A (acetyl-CoA) from the mitochondria to the cytosolic side of the endoplasmic reticulum (ER) (Figure 1). Here, lanosterol synthase, an ER-resident enzyme, converts it into lanosterol, the first sterol and a biologically active molecule in the cholesterol biosynthesis pathway. This pathway then splits into two routes: the Bloch pathway (mainly in astrocytes) and the Kandutsch–Russell (KR) pathway (predominantly in neurons) [84]. Within the cholesterol biosynthesis pathway, most enzyme-encoding genes have a sterol response element (SRE) in their promoter region to which the master transcription factor sterol regulatory element-binding protein 2 (SREBP2) binds, triggering their transcription [85]. In cholesterol-depleted cells, the cholesterol sensor SREBP cleavage-activating protein (SCAP) escorts SREBP2 from the ER to the Golgi. Here, two proteases cleave SREBP2, releasing an active 68 kDa amino-terminal SREBP2 fragment. This fragment then enters the nucleus, binds the SRE in target genes, and activates their transcription, leading to initiation of the cholesterogenic pathway. In the adult mouse brain, astrocytes marked by glial fibrillary acidic protein staining exhibit strong SREBP2 expression under physiological conditions, indicating a primary role in brain cholesterol production. Deletion of *SREBP2* in astrocytes starting from embryonic day 15 results in significant brain size reduction and abnormal behavior, especially in learning and memory tasks [86]. Most mice with SREBP2 knockout in their astrocytes cannot be trained for the Stone T-maze test, a standard memory and spatial learning assessment. This evidence first linked astrocytic cholesterol with cognition. Knockout mice display increased SREBP2 staining in their neurons, suggesting that a loss of SREBP2 and cholesterol synthesis in astrocytes is partially compensated by cholesterol production in the neurons [86]. Despite this compensatory mechanism, the detrimental impact on brain function due to the loss of astrocytic cholesterol production demonstrates that the cellular source of cholesterol production in the brain is not interchangeable, nor can cholesterol levels be adequately compensated. Deletion of *SCAP* in astrocytes, a gene coding for a protein upstream of SREBP, results in a more severe phenotype in mice compared with *SREBP2* knockout mice [87]. This results in the expected downregulation of SREBP-mediated gene expression in astrocytes, leading to reduced phospholipid and cholesterol secretion from astrocytes. Consequently, the mice exhibited lower levels of cholesterol and its intermediates in the brain, decreased brain size and weight from the early postnatal stages, and progressive motor defects. Notably, 60% of these mice died prematurely. The surviving mice exhibited immature synapses, reduced SNAP25 presynaptic protein levels, fewer synaptic vesicles, and impaired synaptic function [39]. These findings further underscore the critical role of astrocytic cholesterol synthesis in maintaining a healthy brain physiology. Cholesterol synthesized in astrocytes is transported to neurons via apolipoprotein E (ApoE)-containing lipoproteins, underscoring the critical role of astrocytes in supporting neuronal health and function [83,85,88].

Astrocytes take up fatty acids from the blood stream through their perivascular process and convert them into energy or store them as lipid droplets (LDs) [70]. These LDs can be mobilized during periods of increased energy demand or stress, providing a readily available energy source for neurons [89,90,91]. Astrocytes, like hepatocytes, can break down fatty acids and produce KBs [44,92,93]. Fatty acids are converted to acetyl-CoA in the mitochondria through beta oxidation (β oxidation), leading to the production of β-hydroxybutyrate (BHB) via a series of enzymatic reactions involving acetyl coenzyme A acetyltransferase (ACAT) and β-hydroxy-3-methylglutaryl coenzyme A (HMG-CoA). Astrocytes and hepatocytes therefore share quite similar fatty acid-oxidizing enzymatic equipment, including the A isoform of carnitine palmitoyl transferase (CPT1A) as a crucial element for the translocation of fatty acids into the mitochondria for β oxidation [92,93]. A recent study highlighted the importance of astrocytic CAPT1A in the overall brain biology and physiology [43] by showing that the astrocyte-selective *CAPT1A* knockout mouse model displays overt cognitive deficits.

Conditions like prolonged fasting, uncontrolled diabetes, and breastfeeding increase circulating BHB and acetoacetate [94]. The liver supplies most KBs to the BBB, which the brain oxidizes when glucose is scarce. KBs enter the brain via the BBB through monocarboxylate transporter 1 (MCT1), which is found in endothelial cells, oligodendrocytes, and astrocytes. Astrocytes, therefore, are not only able to produce KBs but also take up and release BHB [44,70], and this process, by sustaining neuronal activity during periods of prolonged environmental energy shortage, may have been crucial in the evolution of the human brain’s size [95]. The brain adapts to using KBs to maintain synaptic function and structural stability under these conditions. Neurons primarily express the MCT2 isoform, which has high affinity for BHB from endothelial cells and astrocytes [96], and KBs serve as an alternative energy source for neurons, particularly during high energy demand or glucose scarcity [17,70]. Once inside neurons, BHB is converted back into acetyl-CoA, entering the TCA cycle for ATP synthesis [97]. Interestingly, the oxidation of KBs by neurons and oligodendrocytes is three times more efficient than that by astrocytes [47]. KBs also serve as substrates for lipid production, such as myelin [98], and protect myelin-forming oligodendrocytes, reducing axonal damage [99]. KBs can activate intracellular signaling pathways through the posttranslational modification of proteins [100]. MCT2 in neurons is mainly found in mitochondria-rich postsynaptic density structures, indicating KBs’ role in synaptic transmission [101]. Enhanced synaptic activity releases neurotransmitters which stimulate astrocytes to produce lactate and ketones for cellular activity [102]. Glutamate enhances ketogenesis in astrocytes via glutamate transporters [103], and ketones can modulate neuronal firing by opening ATP-sensitive calcium channels [104]. This regulatory role of ketones in neuronal activity may explain the efficacy of a ketogenic diet in treating epilepsy and other neurological disorders.

Astrocytes, due to their adaptive nature and strategic location, interfacing with both blood vessels and neurons, are key in integrating and transmitting peripheral signals to central neuronal networks in the brain [105,106,107]. This function is essential for the neuroendocrine regulation of whole-body metabolism. For example, hypothalamic astrocytes are crucial in sensing various metabolic signals from the periphery, including hormones and nutrients such as leptin [108], ghrelin [109], insulin, glucose [110], fatty acids [111,112], and amino acids [10,113]. In collaboration with neurons, they orchestrate specific metabolic responses [114]. For example, leptin, an anorexigenic hormone produced and secreted by adipose tissue, signals satiety to the brain [115,116] and exerts rapid synaptic modulatory effects in the arcuate nucleus of the hypothalamus (ARH) [117]. Astrocytes express leptin receptors (LepRs) (primarily the long isoform LepRb) [108], which are essential for integrating peripheral leptin signals in the brain [115,118]. Deletion of the *LepRs* in GFAP-expressing astroglial cells can lead to an altered glial morphology, reduced glial coverage, changes in the synaptic input to pro-opiomelanocortin (POMC) and agouti-related peptide (AgRP) neurons, and a diminished anorexic response to leptin [108]. This synaptic modulation mediated by leptin is partly controlled by astrocytes which ensheathe the neurons in the hypothalamic feeding center [108,119,120].

Interestingly, neurons and astrocytes have different but complementary metabolic profiles, leading to extensive metabolic cooperativity. While both cell types can oxidize glucose and lactate, neurons rely more on oxidative metabolism due to their higher energy requirements. Neurons can efficiently use lactate as an energy substrate, sometimes preferring it over glucose. This preference is partly due to the low production of fructose-2,6-bisphosphate (fructose-2,6-P2) in neurons, resulting from constant degradation of the enzyme 6-phosphofructose-2-kinase/fructose-2,6-bisphosphatase-3 (Pfkfb3). This enzyme is highly expressed in astrocytes but virtually absent in neurons, leading to a slower glycolytic rate in neurons and a higher glycolytic rate in astrocytes [38,121,122].

Furthermore, astrocytes play an essential role in maintaining the balance of key metabolites in the brain. They are involved in the transport and metabolism of various energy substrates, such as lactate, pyruvate, glutamate, and glutamine, which are crucial for neuronal function [123,124,125]. The compartmentalization of cerebral metabolism necessitates complex intercellular trafficking of these metabolites, highlighting the critical role of astrocytes in brain energy homeostasis [38,121,122]. These inherent metabolic properties of astrocytes underscore their critical position in the CNS, especially under physiological conditions, and form the basis for understanding the metabolic changes in reactive astrocytes.

## 3. Metabolic Reprogramming of Astrocytes in Pathological Conditions

**Figure 2:**
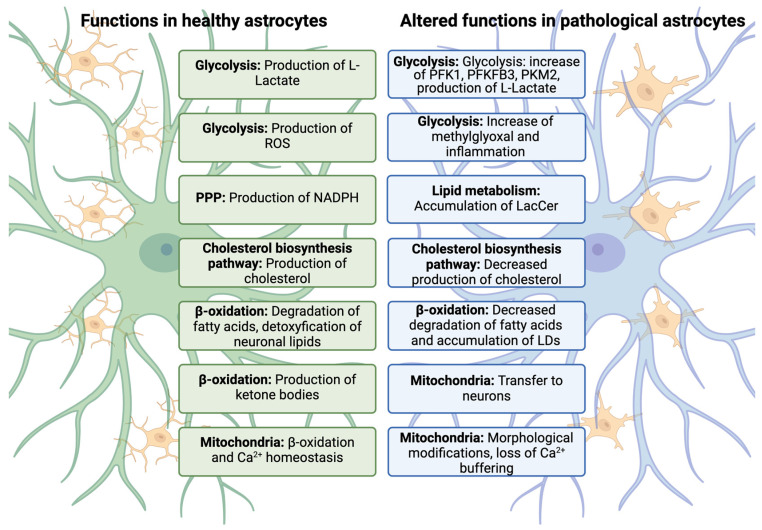
The figure contrasts the normal metabolic functions of astrocytes (depicted on the left in green) with the metabolic alterations observed in various sub-states of reactive astrocytes (depicted on the right in light blue). These changes in reactive astrocytes were identified through experimental studies on inflammation and neurodegenerative diseases. It is important to note that the depicted functions do not correspond to a single astrocyte type, and reactive astrocytes may still retain some physiological functions despite their altered states. Figure modified from [128].

Astrocytes exhibit significant morphological, molecular, and functional changes in response to CNS pathologies such as neurotrauma, stroke, brain hemorrhage, infections, epilepsy, and Alzheimer’s disease (AD) [126]. They are notably abundant in regions with high amyloid-β (Aβ) or tau pathology in postmortem AD brains and contribute to neuronal damage by releasing cytokines, inflammatory factors, nitric oxide (NO), and reactive oxygen species (ROS) [29,127]. Indeed, under pathological conditions, astrocytes respond rapidly to brain injuries by first morphologically changing their processes to become more ramified and larger, with elongated processes. These are referred to as “reactive astrocytes” [126,128]. Decades of studies, particularly those utilizing in vivo imaging technologies and bulk or single-cell transcriptome and proteomic studies, have revealed that reactive astrocytes undergo molecular, functional, and metabolic changes alongside their well-characterized morphological changes. Despite these advancements, the detailed molecular and cellular features of reactive astrocytes remain under investigation. This is mainly because the pathological contexts in which astrocyte reactivity occurs can vary markedly and may be sporadic or genetically mediated, acute, or chronic due to systemic pathologies, specific injuries to or diseases of the CNS, or detrimental experimental manipulations. The initial binary characterization of reactive astrocytes into good versus bad, neurotoxic versus neuroprotective, or A1 versus A2 [129] is no longer accepted. Indeed, recent studies challenged the binary classification of reactive astrocytes into “A1” and “A2”. For example, some research on human and mouse models of HD did not find a distinct A1 signature [130]. Additionally, activation of the unfolded protein response in astrocytes can lead to a neurotoxic state which does not align with the A1 and A2 framework [131]. Current debate [126] emphasizes the complex and heterogeneous nature of reactive astrocytes. Astrocytes respond differently based on the pathology and environmental context, exhibiting a range of reactive states rather than fitting neatly into the A1 and A2 dichotomy. These findings suggest that reactive astrocytes in different CNS diseases can exhibit distinct characteristics and roles, further complicating their classification. Therefore, a more nuanced understanding of astrocyte heterogeneity is necessary, moving beyond the binary A1 and A2 descriptors to better grasp their diverse functions and potential therapeutic implications. In recent years, using human samples and sophisticated animal models, several states of reactive astrocytes with distinct molecular and functional phenotypes have been characterized, including the so-called “neuroinflammatory astrocytes”, “neurotoxic reactive astrocytes”, and “disease-associated astrocytes” [132,133,134]. We now understand that depending on the specific model or disease, these reactive astrocytes states could be either “neuroprotective” or “neurotoxic”, and multiple states or reactive astrocytes may coexist with temporal and spatial heterogeneity [126,135,136]. In some pathological states, the “bad” reactive astrocytes form rapidly after CNS injury, and they actively participate in inflammatory processes alongside microglial cells, ultimately killing neurons and oligodendrocytes. This phenotype is prevalent in neurodegenerative diseases such as Huntington’s disease (HD), Parkinson’s disease (PD), amyotrophic lateral sclerosis (ALS), multiple sclerosis (MS), and AIDS dementia complex [29,129] (Figure 2). Indeed, pro-inflammatory cytokine tumor necrosis factor (TNF) released by activated microglia boosts glutamate production [29,137], which in turn triggers excitotoxicity and Ca^2+^ signaling. Ca^2+^ elevation in astrocytes releases glutamate, ATP, and γ-aminobutyric acid (GABA) [29,138,139,140]. Moreover, excessive GABA and ATP release by reactive astrocytes has been linked to disease pathogenesis in AD models [141,142]. Conversely, reactive astrocytes can also assume a neuroprotective phenotype, promoting motor recovery by supporting vascular repair in ischemic stroke models and assisting in clearing cellular debris and protein aggregates [143]. Depletion of reactive astrocytes exacerbates disease severity in models of experimental autoimmune encephalomyelitis (EAE) and spinal cord injury (SCI) [144,145], indicating their protective role. Transitioning from a “resting” to “reactive” state, astrocytes undergo significant metabolic adaptations to meet increased energy demands and alter their functionality in response to injury or stress [146,147]. These changes are crucial for their diverse roles in the brain’s defense mechanisms against injury, inflammation, and neurodegenerative diseases. This section explores the evolving roles of glucose, lipid, and mitochondria metabolism in reactive astrocytes, with a particular emphasis on the dual functions of glycolytic and lipid enzymes in shaping the biological characteristics of reactive astrocytes in neurological diseases.

### 3.1. Alteration of Glycolytic Pathways in Reactive Astrocytes

In their reactive state, astrocytes upregulate glycolysis to meet the increased energy demands associated with their response to injuries. This heightened glycolytic activity not only ensures their survival during hypoxia for up to one hour but also supports various critical functions [37]. For instance, increased glycolysis facilitates the production of L-lactate, which serves as an energy substrate for neighboring neurons, aiding their survival during hypoxic conditions [38,58]. Enhanced glycolysis in astrocytes has been shown to significantly improve cognition, promote neuronal survival, and facilitate axon growth in the 5×FAD mouse model of AD [59]. Conversely, impaired astrocytic glycolysis can lead to increased accumulation of amyloid-beta (Aβ) within and around astrocytes, rendering these cells more vulnerable to Aβ toxicity [148]. Furthermore, L-lactate aids neuronal energy metabolism in response to heightened brain activity [37,38]. However, excessive glycolysis can also exacerbate inflammation, leading to astrocyte activation and contributing to neuroinflammatory responses in some brain diseases [149,150]. This mechanism is elusive, since previous reports have shown decreased astrocytic glycolysis when astrocytes are exposed to proinflammatory stimuli, such as interleukin 1alpha or TNF alpha [151]. Subsequent decreased glycogen and lactate levels might be related to a neuroprotective effect exerted by astrocytes, although such a mechanism was shown in primary cultures [152,153]. On the other hand, one methylglyoxal pathway has been recently proposed as a mechanism linking excessive glycolysis and neuroinflammation [154]. Methylglyoxal is a byproduct of cell glycolysis, known to be involved in a number of pathological and neurodegenerative disorders [155,156]. Since methylglyoxal is highly toxic and cell permeant, increased glycolysis coupled with a dysfunctional detoxification pathway might likely link glycolysis and neuroinflammation in pathologies like AD [157] or diabetes [158].

Inflammatory responses often associated to pathological states involve the coordination of various participants, including innate immune cells like neutrophils and macrophages, as well as brain cells such as astrocytes and microglia. When immune cells and microglia are activated to initiate an inflammatory response, they undergo metabolic reprogramming by increasing glycolysis. This enables faster production of ATP in activated microglia [159], allowing rapid metabolism for cell growth and the production of cytokines and reactive oxygen species [160]. This metabolic switch is pivotal in maintaining homeostatic functions and is critical for progression and repair mechanisms upon CNS injury and neurodegeneration [161]. It also determines the fate of microglial cells and, by maintaining their ability to release cytokines, subsequently influences the fate of astrocytes [29], significantly impacting the overall dynamics of the inflammatory response [162,163,164]. Thus, the upregulation of glycolysis is considered a double-edged sword in regulating the development of neurological diseases, warranting further investigation into the underlying mechanisms.

Central to regulating glycolysis in reactive astrocytes is the enzyme 6-phosphofructo-2-kinase/fructose-2,6-bisphosphatase-3 (PFKFB3), which is highly abundant in mature astrocytes [56,165]. PFKFB3 accelerates the production of fructose-2,6-bisphosphate (fructose-2,6-P2), a potent activator of the glycolytic enzyme PFK1. This enzyme is crucial for maintaining the glycolytic phenotype of reactive astrocytes, contributing to their energy production and functional responses [166]. Experiments using in vitro models of oxygen-glucose deprivation and reoxygenation have demonstrated that heightened PFKFB3 and PFK1 expression markedly enhances reactive astrocyte proliferation and lactate release [166], suggesting a protective role for increased glycolysis. However, increased PFKFB3 activity is also associated with significant reactive astrogliosis surrounding Aβ plaques in mouse models of AD and pharmacological inhibition or molecular downregulation of PFKFB3, resulting in increased accumulation of Aβ within and around astrocytes and greater vulnerability of these cells to Aβ toxicity. This indicates that PFKFB3-mediated glycolysis may play a role in AD development [148]. These studies underscore PFKFB3’s necessity and sufficiency in maintaining the glycolytic phenotype of astrocytes, shedding light on the bioenergetic mechanisms within reactive astrocytes.

In the reactive state, astrocytes also express pyruvate kinase isoform M2 (PKM2), which differs from the predominant neuronal isoform PKM1 [167,168]. PKM2 translocates to the nucleus, where it regulates cell cycle-related proteins and signaling in response to injury [169]. The dynamic behavior of PKM2, switching between monomeric or dimeric and tetrameric forms, influences its enzymatic activity and its ability to regulate various cellular processes, including gene expression and protein kinase activity [169]. For example, nuclear PKM2 stabilizes the transcription factor hypoxia-inducible factor 1-α (HIF-1α), coordinating the Warburg effect by inducing glucose transporters and glycolytic enzymes such as aldolase A [167]. PKM2-mediated aerobic glycolysis also prompts the release of high-mobility group box 1 (HMGB1), which inhibits PKM2, blocking glucose-driven aerobic respiration and forcing astrocytes to increase glycolysis to maintain energy production, which contributes to the pathological processes of various brain diseases [170,171].

### 3.2. Lipid Metabolism Alteration in Reactive Astrocytes

In addition to glycolytic changes, lipid metabolism undergoes adaptations in reactive astrocytes. Fatty acid oxidation (FAO) provides approximately 20% of oxidative energy production [42] and is essential for energy generation, especially during fasting, prolonged exercise, or low-carbohydrate intake periods [172]. FAO efficiently converts stored fatty acids into acetyl-CoA, which enters the citric acid cycle and electron transport chain to produce ATP, the primary energy currency of cells [173]. Given the crucial role of FAO in meeting energy demands, it has been implicated in various pathological states, primarily related to an inadequate energy supply or the accumulation of toxic metabolites. In the brain, FAO primarily occurs in astrocytes, as demonstrated by RNA profiling and co-localization studies [56,92,93,174,175]. An increase in FAO in aging astrocytes may compensate for decreased glucose utilization, indicating a protective mechanism against neurodegenerative diseases [176,177]. LD generation, typically intended to provide fuel for β oxidation, plays a pivotal role in this process. For example, toxic fatty acids produced in hyperactive neurons may be transferred to astrocytic LDs, generating energy through mitochondrial β oxidation [41]. The brain relies heavily on astrocytic mitochondrial β oxidation and oxidative phosphorylation to break down fatty acids and preserve the lipid balance. Aberrant astrocytic oxidative phosphorylation, leading to LD accumulation, results in neurodegeneration which mimics the key features of AD, including synaptic loss, neuroinflammation, demyelination, and cognitive impairments [178]. LDs are highly dynamic organelles crucial to cellular signaling, energy metabolism, and immune activity. They consist of a hydrophobic core of neutral storage lipids, such as triacylglycerides and cholesteryl esters, surrounded by a monolayer of phospholipids and coated with a variety of regulatory proteins [179]. Alois Alzheimer first described lipid saccules as a characteristic of the AD brain [180]. More recently, LDs have also been shown to accumulate in the aging brain [181,182]. Polymorphisms in the APOE gene represent the strongest genetic risk factor for AD (APOE4) and the strongest genetic protective factor (APOE2) compared with the neutral variant (APOE3) [183,184]. Furthermore, secreted ApoE has been shown to bind amyloid beta (Aβ), regulating its compaction and clearance and thus influencing the amyloid plaque pathology [184]. Recent discoveries have demonstrated that APOE4 disrupts the cellular lipidome in human iPSC-derived astrocytes by increasing unsaturated fatty acids and promoting the accumulation of LDs [185]. This disruption directly decreases FAO in astrocytes and causes LD accumulation in neurons [186]. Finally, Windham et al. [187] identified a novel intracellular role for ApoE in shaping LDs through its localization at the cytosolic leaflets of these organelles. Although ApoE had previously been detected on LD surfaces [188,189], these early observations did not receive significant attention with the respect to its functional role. Windham et al. provided evidence that ApoE is present on LDs and plays a crucial role in regulating their size and triglyceride metabolism, thus providing further insight into the complex role ApoE plays in the brain and opening several important research routes to understanding and eventually treating ApoE4-dependent AD.

In patients with temporal lobe epilepsy and mouse models of epilepsy, excessive lipid accumulation in astrocytes leads to the formation of lipid-accumulated reactive astrocytes, termed “LARAs”. LARAs represent a new reactive astrocyte subtype found in mouse models of epilepsy and brain tissues from patients characterized by elevated APOE expression, suggesting that targeting LARAs through lipid transport and metabolism interventions could offer new therapeutic options for drug-resistant temporal lobe epilepsy [190]. Because LARAs are formed by neuron-astrocyte lipid transfer, they may also exist in other diseases associated with neuronal hyperactivity, such as traumatic brain injuries. The mechanism of protection, exercised by FAO, has been found to be crucial in protecting neurons against damage induced by ischemic stroke, highlighting the neuroprotective role of increased astrocytic energy production mediated by heightened FAO [191]. However, findings from HD models suggest that groups of reactive astrocytes transitioning from glycolysis to FAO may also adopt a neurotoxic phenotype, contributing to increased damage induced by reactive oxygen species (ROS) [192]. In the context of HD, evidence suggests that early, region-specific neuronal susceptibility in HdhQ (150/150) animals arises from metabolic reprogramming in their astrocytes. In the HD brain, glucose levels are notably low, prompting astrocytes to energetically adapt by reprogramming their mitochondria to utilize endogenous, non-glycolytic metabolites as alternative fuels. However, this metabolic reprogramming varies by brain region, with astrocytes in vulnerable and resistant areas employing different adaptation pathways. In the vulnerable striatum, which is enriched in fatty acids, astrocyte mitochondria switch to FAO. This switch helps sustain energy production but comes at the cost of elevated reactive oxygen species, leading to oxidative damage. Conversely, astrocytes in the resistant cerebellum utilize amino acid precursors to regenerate glucose, thereby avoiding ROS-induced damage. The striatum’s higher susceptibility to toxicity can be attributed to this metabolic shift which, while maintaining energy production during metabolic crises, ultimately results in greater oxidative damage compared with the cerebellum. This suggests that metabolic reprogramming, though a common strategy to sustain energy production, may lead to region-specific vulnerabilities due to the different fuel sources and the associated risks of damage.

Lipids exert pronounced effects on inflammation in the context of autoimmunity and microbial infections, acting either as targets or regulators of the immune response [193,194,195]. In a study performed with mice models of multiple sclerosis (MS), the authors found that lactosylceramide (LacCer), synthesized by β-1,4-galactosyltransferase 6 (B4GALT6), is upregulated in the CNS of mice during chronic experimental autoimmune encephalomyelitis [144]. The accumulation of LacCer in reactive astrocytes controls the recruitment and activation of microglia and infiltrating monocytes by regulating the production of chemokine CCL2. This indicates that reactive astrocytes may control processes which drive CNS inflammation and neurodegeneration. Consequently, the modulation of glycolipid synthesis represents a potential therapeutic approach for MS and other neurological disorders in which reactive astrocytes accumulate LacCer.

Fatty acids are transported by fatty acid-binding proteins (FABPs), a family of lipid chaperones involved in systemic metabolic regulation through various lipid signaling pathways [196]. FABP7, the predominant isoform in the brain, is mainly expressed in astrocytes [56] and influences dendritic morphology, synaptic transmission, and neuroprotection by aiding in LDs formation to store potentially toxic fatty acids [197]. While the precise roles of FABPs in regulating astrocyte function are not fully understood, preliminary studies indicate that astrocytic FABP7 is crucial for both physiological and pathological brain functions. For instance, FABP7 is vital for reactive astrocyte proliferation following CNS injury and modulates astrocyte responses to external stimuli by regulating lipid raft function [198], suggesting a neuroprotective role. However, some studies, such as that by Killoy et al., have shown that FABP7 can induce a neurotoxic phenotype in astrocytes, contributing to motor neuron toxicity in ALS [199]. Despite inconsistencies in the findings, these studies offer preliminary insights into the role of lipid metabolism in astrocyte heterogeneity. Indeed, astrocytes release various lipids in response to different stimuli, resulting in diverse effects. For example, astrocytes exposed to lipopolysaccharide (LPS) release polyunsaturated fatty acids like docosahexaenoic acid (DHA) [200], which can reduce the proinflammatory response in a mouse model of a spinal cord injury (SCI). Conversely, neurotoxic astrocytes triggered by interleukin-1α (IL-1α), tumor necrosis factor (TNF), and complement component 1q (C1q) release saturated lipids in apolipoprotein E (APOE) and apolipoprotein J (APOJ) lipoparticles, which have been shown to mediate astrocyte-induced toxicity in the CNS [201]. Recent studies have highlighted that cholesterol production in astrocytes is mediated by pro-inflammatory cytokines such as TNF-α [202]. These cytokines induce cholesterol synthesis in astrocytes, which then release the cholesterol for uptake by immune cells. This process leads to the clustering of proinflammatory receptors in lipid rafts, perpetuating the inflammatory signal. Knockout of cholesterol synthesis in astrocytes blocks the production of inflammatory cytokines, thus influencing the escalation and resolution of chronic neuroinflammation [202]. At a cellular level, there is a dynamic interplay between astrocytes and microglia in neuroinflammation. Microglia, the primary immune cells of the brain, release cytokines and factors which activate astrocytes, while astrocytes, the primary support cells for neurons, release both pro- and anti-inflammatory factors affecting microglia. Astrocytes are responsible for producing the majority of the cholesterol in the brain, which is distinct from the peripheral cholesterol obtained from the liver and one’s diet. In vivo RNA profiling of astrocytes from both mouse and human brains using bulk sequencing and single-cell sequencing has shown a significant enrichment of SREBP2 and 12 other cholesterol biosynthesis genes [56,174,175,203,204]. This astrocytic cholesterol production plays a crucial role in neuroinflammation, as evidenced by studies in AD mouse models, where the reduction of neuroinflammation was observed following peripheral injection of lipopolysaccharide (LPS) [202].

The transcriptional profile indicating upregulation of SREBP2 in astrocytes under physiological conditions [56,174,175,203,204] is significantly altered in many pathological conditions. In purified astrocytes from mouse models of HD, multiple sclerosis models, and aged mice, the transcription of SREBP2-regulated cholesterol biosynthesis genes is significantly reduced [205,206,207,208,209].

Overall, these findings suggest that astrocytic lipid metabolism is intricate and crucial in maintaining physiological and pathological conditions. By compensating for impaired glucose metabolism and providing substrates for synaptic and myelin membrane synthesis, astrocytic lipid metabolism is implicated in various neurological diseases. While the precise mechanisms by which lipid metabolism regulates reactive astrocyte phenotype switching in CNS diseases remain largely unknown, these novel insights highlight the potential of targeting astrocytic lipid metabolism as a therapeutic pathway and a new research avenue for neurological disorders.

### 3.3. Mitochondria Metabolism Alteration in Reactive Astrocytes

Mitochondria in astrocytes are crucial for oxidizing glucose and lipids, thereby generating the ATP necessary for cellular energy. Interestingly, impaired mitochondrial function under physiological conditions does not significantly affect astrocyte survival [210]. However, a series of new studies highlighted that mitochondria in astrocytes are vital for both astrocytic operations and the health of neighboring neurons. For instance, astrocyte death has been observed within a day following focal ischemia, potentially due to mitochondrial swelling [211,212]. Additionally, astrocytes exhibit high expression of the molybdenum cofactor-containing mitochondrial enzyme sulfite oxidase, which can catalyze nitrite reduction and nitric oxide (NO) production in hypoxia. This pathway is necessary during hypoxia to orchestrate the cerebrovascular response [213]. These data identify astrocyte mitochondria as brain oxygen sensors which regulate cerebral blood flow during hypoxia through the release of nitric oxide. Mitochondrial dysfunctions have been also identified in aged astrocytes of the human cerebral cortex [214] and in reactive astrocytes, which significantly reduced the generation of new astrocytes and increased neuronal cell death in the perilesional area of ischemic stroke [215]. Timely activation of autophagy is critical for restoring tubular mitochondrial structures, which is essential for orchestrating metabolic changes in astrocytes and for maintaining cell survival during proinflammatory responses [52,211]. Furthermore, acute injury and BBB disruption trigger the formation of a distinct, mitochondria-rich compartment in the astrocytic endfeet, which plays a crucial role in vascular remodeling [53]. Authors, by using integrated imaging techniques, have shown that this mitochondrial clustering is an adaptive mechanism driven by mitochondrial fusion dynamics. When Mitofusin 2 (Mfn2) was specifically deleted in astrocytes, it led to a reduction in perivascular mitochondrial clustering and disrupted the contact sites between mitochondria and the endoplasmic reticulum.

In the last decade, a number of studies have documented the transfer of astrocytic cytosolic mitochondria to other cells. For example, the transfer of healthy astrocytic mitochondria into adjacent neurons has been shown to provide neuroprotection in models of transient focal cerebral ischemia in mice [212]. These findings suggest that healthy mitochondria in astrocytes are beneficial for neurological health, as fragmented mitochondria can trigger a harmful astrocytic response and propagate inflammatory neurodegeneration [216]. Furthermore, stimulating mitochondrial ATP production in astrocytes has demonstrated neuroprotective effects in mouse models of cerebral ischemic stroke [217] and depression [218]. While the complete understanding of how mitochondrial dynamics influence reactive astrocyte phenotypes remains elusive, it is clear that maintaining mitochondrial health is critical for preserving brain energetics and may offer therapeutic benefits for various neurological diseases.

## 4. Conclusions 

The metabolic reprogramming of astrocytes plays a crucial role in the pathophysiology of neurodegenerative diseases. Astrocytes, known for their form, functional diversity, and remarkable adaptive plasticity, support neuronal function through glycolysis, the pentose phosphate pathway, and lipid metabolism, ensuring a balanced energy supply and protection against oxidative stress. However, in pathological states, astrocytes undergo significant metabolic changes to meet increased energy demands and to support their reactive functions.

Reactive astrocytes upregulate glycolysis, enhancing the lactate production essential for neuronal energy metabolism and survival under stress conditions. This metabolic shift, while protective in acute settings, can contribute to chronic inflammation and exacerbate disease progression in conditions like AD and HD. Furthermore, changes in lipid metabolism, including increased fatty acid oxidation and LDs formation, highlight the dual role of astrocytes in neuroprotection and potential neurotoxicity.

Despite considerable efforts to understand the morphological and functional diversity of astrocytes in developmental and diseased brains, the mechanisms underlying the regulation of metabolic alterations and reactive astrocyte heterogeneity remain largely unknown [219]. The metabolic diversity of astrocytes appears to be a key factor driving their heterogeneity in response to CNS diseases, potentially leading to improved strategies to combat neurological disorders. However, linking their morphological diversity to functional and metabolic diversity remains challenging [220].

To systematically document and interrogate astrocyte heterogeneity in vivo, advanced tools for molecular, genetic, morphological, and physiological assessments have been developed or adapted from their original neuronal studies [221,222]. These tools are crucial for exploring several unresolved issues regarding astrocyte heterogeneity, such as the variability of astrocyte metabolism in different brain microenvironments, the types and mechanisms determining astrocyte heterogeneity, and the mechanistic links between reactive astrocytes’ heterogeneity, function, and metabolism under normal and CNS disease conditions.

Understanding the initiation mechanisms of reactive astrocyte heterogeneity in CNS disorders and their evolutionary conservation between humans and rodents is vital. Additionally, exploring how metabolic changes in non-astrocyte nerve cells affect reactive astrocytes’ metabolic and functional heterogeneity under CNS disease conditions is essential. Improved strategies and cutting-edge technologies like spatial metabolomics and single-cell metabolomics may help decipher the heterogeneity of reactive astrocytes and their mechanistic link between function and metabolism, providing crucial insights for enhancing neuroprotection and the treatment of neurological diseases.

## 5. Future Directions and Key Questions in Astrocyte Metabolism Research

Astrocytes play a critical role in neurodegenerative diseases, yet many aspects of their metabolic contributions remain unresolved. Future research must address several key questions to uncover the complexities of astrocyte metabolism and its impact on disease progression.

How do metabolic changes in astrocytes contribute to the progression of neurodegenerative diseases?

What specific pathways in astrocyte metabolism are most affected during different stages of neurodegenerative diseases?

How can we distinguish between the protective and detrimental effects of astrocyte metabolic reprogramming in neurodegenerative conditions?

What are the potential therapeutic targets within astrocyte metabolic pathways for the treatment of neurodegenerative diseases?

How do reactive astrocytes’ metabolic changes influence neuronal survival and function in diseased brains?

How do interactions between astrocytes and other glial cells or neurons affect overall brain metabolism in the context of neurodegenerative diseases?

What role does lipid metabolism play in the dual function of astrocytes in neuroprotection and potential neurotoxicity?

By addressing these questions, future research can pave the way for new therapeutic strategies targeting astrocyte metabolism and heterogeneity, ultimately improving the treatment outcomes for various neurodegenerative diseases.
